# Revolutionizing Cardiac Anesthesia: A Comprehensive Review of Contemporary Approaches Outside the Operating Room

**DOI:** 10.7759/cureus.55611

**Published:** 2024-03-05

**Authors:** Nandha Kumar Durai Samy, Karuna Taksande

**Affiliations:** 1 Anaesthesiology, Jawaharlal Nehru Medical College, Wardha, IND

**Keywords:** patient-centered care, hybrid operating rooms, interventional cardiology, contemporary approaches, non-operating room environments, cardiac anesthesia

## Abstract

This review article provides a comprehensive examination of the evolution of cardiac anesthesia, emphasizing contemporary approaches beyond the traditional operating room (OR) setting. Tracing the historical roots of cardiac anesthesia from its inception in the mid-20th century, the narrative explores the significant paradigm shift driven by technological advancements and changing procedural approaches. The review highlights the emergence of non-OR environments, such as hybrid operating rooms, catheterization laboratories, and electrophysiology labs, as integral spaces for cardiac interventions. Key findings underscore the importance of patient selection, preoperative assessment, and specialized anesthetic management in optimizing outcomes. Implications for the future of cardiac anesthesia include the potential for enhanced patient-centered care, reduced complications, and improved resource utilization through the integration of advanced technologies. The call to action involves encouraging ongoing research and fostering collaboration among healthcare professionals to refine protocols further, address challenges, and propel the field toward continued innovation in contemporary cardiac interventions.

## Introduction and background

Cardiac anesthesia has long been a critical component of perioperative care, which is pivotal in ensuring patient safety and optimal outcomes during cardiac surgical procedures. Traditionally confined to the operating room (OR), cardiac anesthesia has witnessed a paradigm shift in recent years, with an increasing emphasis on interventions and procedures conducted outside the traditional surgical setting. This shift has been driven by advancements in technology, changes in procedural approaches, and a growing recognition of the potential benefits of non-OR environments for certain cardiac interventions [1]. The historical roots of cardiac anesthesia trace back to the mid-20th century, marked by milestone achievements such as the introduction of cardiopulmonary bypass and the development of specialized techniques to manage the unique physiological challenges associated with cardiac surgery. Over the years, the discipline has evolved to encompass a broader spectrum of interventions, extending beyond the confines of the operating room [2].

Contemporary approaches to cardiac anesthesia outside the operating room (OR) carry profound significance in cardiovascular care. The expansion of interventions to non-OR settings, including hybrid operating rooms, catheterization laboratories, and electrophysiology labs, reflects a strategic response to the evolving landscape of cardiac medicine. These alternative environments offer unique advantages, such as real-time imaging capabilities, specialized equipment, and the ability to perform various procedures ranging from minimally invasive interventions to complex electrophysiological studies [3]. The significance of these contemporary approaches is underscored by their potential to enhance patient outcomes, reduce procedural complications, and optimize resource utilization. By leveraging the capabilities of non-OR settings, healthcare providers can tailor interventions to individual patient needs, fostering a more personalized and efficient approach to cardiac care [4].

The primary objective of this comprehensive review is to meticulously examine and integrate the current state of contemporary approaches to cardiac anesthesia beyond the confines of the OR. By thoroughly investigating the historical context, tracing the evolution of techniques and technologies, and evaluating their impact on patient care, this review aims to thoroughly understand the challenges and opportunities inherent in non-OR cardiac interventions. The scope of this review encompasses diverse topics, including the justification for performing cardiac interventions outside the OR, criteria for patient selection, anesthetic management strategies in non-OR environments, considerations for complications and contingency planning, initiatives for training and education, and a forward-looking exploration of future directions and innovations in the field. Through a systematic exploration of these aspects, this review aspires to make a meaningful contribution to the broader discussion on the progression of cardiac anesthesia and its relevance to contemporary cardiovascular care.

## Review

Traditional cardiac anesthesia in the operating room

Overview of Standard Cardiac Anesthesia Procedures

In traditional cardiac anesthesia within the OR, general anesthesia is the standard practice, characterized by inducing complete anesthesia affecting the entire body, resulting in loss of consciousness, analgesia, amnesia, and muscle relaxation. The selection of anesthetic agents and their combinations is tailored to the specific pathophysiological state of each patient. Widely used opioids in cardiac surgery, including fentanyl, sufentanil, and remifentanil, are employed in moderate doses to mitigate adrenergic responses. Muscle relaxants prevent patient movement, and potent inhaled anesthetics enhance the relaxant effect, safeguard the heart, and modify hemodynamic perturbations [5,6]. There is a notable shift of interest in cardiac surgery toward chest wall blocks, a regional anesthesia technique gaining prominence due to its associated benefits. Unlike neuraxial techniques, which have sparked controversy owing to concerns about hemodynamic instability and the risk of spinal hematoma, chest wall blocks are emerging as an attractive alternative. These blocks, including the pectoralis fascial, serratus anterior plane, erector spinae plane, and paravertebral blocks, are gaining attention for their ability to avoid significant respiratory depression [7]. This evolution in anesthesia approaches is particularly noteworthy in cardiac surgery, where mitigating the risk of bleeding and minimizing sympathetic blockade are crucial considerations. The conventional setup for cardiac anesthesia in the OR typically involves using various equipment, encompassing IV poles, monitoring devices, and anesthesia delivery systems [8]. However, the growing interest in chest wall blocks represents a significant development, reflecting a contemporary exploration of regional anesthesia techniques in pursuing enhanced patient outcomes in cardiac surgical procedures.

Limitations and Challenges in the Operating Room Setting

The OR environment's cleanliness and infection control measures distinguish it from the wards or ICU, thereby mitigating infection risks. Despite these advantages, the OR does present several challenges that can impact patient care and outcomes. These challenges encompass constrained space, elevated noise levels, time constraints, breakdowns in communication, restricted access to resources, compromised visibility, and heightened patient anxiety [9]. Additionally, contemporary ORs frequently contend with inefficiency and overcrowding, leading to prolonged and variable turnover times between cases [10]. Navigating the complexities within the OR proves challenging, given its dynamic and resource-constrained nature, necessitating specific technical and cognitive skills from the surgical team [11]. Further complicating matters are ineffective relationships with the supply chain, lack of electronic access to real-time patient imaging and health information, and conflicts between surgeon demands and personalities that may clash with nursing staff [12]. Effectively addressing these limitations and challenges demands adept management and coordination of resources, along with incorporating new technologies and processes to enhance workflows, safety protocols, and overall performance within the OR.

Evolution of cardiac anesthesia beyond the operating room

Historical Perspective on Out-of-OR Cardiac Procedures

The history of cardiac anesthesia and surgery has evolved significantly throughout the past century. Modern cardiac surgery traces its origins to the late 1930s when interventions initially focused on intrathoracic blood vessels rather than direct procedures on the heart [6]. The expansion of surgical techniques targeting the heart spurred the concurrent development of individuals, methodologies, and pharmacological approaches to support surgeons, with a notable contribution from anesthesiologists [6]. Compared to other disciplines, the history of cardiothoracic anesthesia is relatively brief, commencing toward the end of the nineteenth century [13]. The initial challenge of pneumothorax was overcome with the introduction of tracheal intubation and lung isolation techniques advanced with the advent of bronchial blockers, double-lumen tracheal tubes, and the refinement of fiber optic bronchoscopy [13]. The advent of cardiopulmonary bypass marked a pivotal moment in cardiac surgery, though it presented new challenges, including the maintenance of anesthesia during procedures [13]. The application of various anesthetic drugs to cardiac anesthesia has been extensive, emphasizing the importance of aligning patient pathophysiology with pharmacology for optimal care [6]. The evolution of anesthesia for cardiothoracic surgery has been nothing short of dramatic, with the specialty emerging from the shadow of cardiothoracic surgeons to become an independent, self-sufficient, and fiercely competitive field [14]. Presently, cardiac anesthesiology is a rapidly advancing discipline, incorporating technological and procedural innovations to continually improve patient care and outcomes [6,14,15].

Advancements in Technology and Techniques

Recently, the field of anesthesia, particularly cardiac anesthesia, has undergone a revolutionary transformation propelled by notable advancements in technology and techniques. A key development is the automation of anesthetic delivery, wherein systems featuring independent closed loops for hypnosis, analgesia, and fluid management have become increasingly scalable and efficient, exemplifying a paradigm shift in patient care [16]. The evolution of monitoring technology has been equally impactful, with the analysis of the peripheral arterial pressure waveform now enabling cardiac output monitoring. Furthermore, truly noninvasive assessments of cardiac output, facilitated by innovative methods using a blood pressure cuff, represent a significant stride forward [16]. Artificial intelligence (AI) has emerged as a transformative force in anesthesia, ushering in novel approaches to patient care, real-time monitoring, and data-driven decision-making [17,18]. Integrating AI algorithms into the field promises to enhance precision and efficiency, ultimately optimizing anesthesia administration and patient outcomes. Moreover, the advent of perioperative imaging, including 3D transesophageal echocardiography, alongside the introduction of new devices and drugs, further underscores the dynamic landscape of contemporary cardiac anesthesia [17]. The assimilation of these cutting-edge advancements has translated into tangible improvements in patient outcomes, with discernible reductions in morbidity and mortality [17]. As technology continues to evolve, the trajectory of cardiac anesthesia is marked by a commitment to leveraging innovation to improve patient care and overall clinical outcomes.

Shift in Paradigm: Importance of Non-Operating Room Environments

Unfamiliar environment: Operating in non-operating room anesthesia (NORA) environments introduces the challenge of personnel being unfamiliar with established OR protocols. This lack of familiarity may lead to discomfort among the staff, particularly when dealing with patients under anesthesia [19]. The unfamiliarity with the unique aspects of NORA settings can potentially impact the coordination and seamless execution of procedures.

Limited workspace and resources: NORA procedure rooms are often purpose-built for specific medical interventions, resulting in constrained workspace and limited resources compared to the more versatile traditional ORs. This limitation can potentially hinder the efficiency and flexibility required for diverse procedures [20]. The challenge lies in adapting to a confined environment and optimizing available resources to ensure optimal patient care.

Inadequate lighting and temperature regulation: In NORA environments, there may be challenges related to insufficient lighting and inadequate temperature regulation. These factors can directly influence patient care and staff comfort, potentially affecting the precision of medical procedures and the overall well-being of the healthcare team [19]. Striking the right balance between lighting and temperature control becomes imperative for maintaining optimal conditions in these settings.

Remote location: NORA cases are often conducted in remote locations, geographically distant from centralized pharmacies and medical supplies. This geographical distance can pose significant challenges in workflow and patient safety, requiring careful logistical planning and coordination to ensure the availability of essential resources [19]. Overcoming the hurdles associated with remote locations is crucial for maintaining the quality and safety of medical interventions.

Noisy environments: NORA environments are prone to increased noise levels, disrupting patient monitoring and hindering effective communication among healthcare professionals. This noise interference may compromise the precision of medical procedures and potentially impact patient outcomes [19]. Mitigating noise disruptions becomes critical in managing NORA environments to ensure a conducive atmosphere for patient care and effective teamwork among healthcare providers.

Emergency procedures: A greater percentage of NORA procedures are performed on an emergency basis, which can further complicate patient care and safety [19]. Despite these challenges, NORA has become an increasingly important aspect of patient care, offering minimally invasive treatments in various fields [20]. To minimize adverse outcomes, anesthesiologists should remain vigilant and familiarize themselves with the NORA environments [20]. Implementing protocols and interdisciplinary teamwork can facilitate safe, efficient, and cost-effective procedural care in NORA settings [19].

Contemporary approaches in cardiac anesthesia

Hybrid Operating Rooms: Integration of Surgical and Interventional Procedures

Enabling new cardiac surgery therapies: The hybrid OR concept has ushered in a new era in cardiac surgery, allowing for the execution of a diverse range of procedures, including minimally invasive surgeries. This capability represents a substantial leap forward in patient care, as these procedures often contribute to improved outcomes and reduced postoperative pain. The hybrid OR is a versatile platform for innovative cardiac surgery therapies that enhance the quality of patient care, ultimately leading to advancements in the field [21].

Space optimization: In smaller interventional suites, anesthesia, and perfusion equipment can create space constraints. The hybrid OR addresses this challenge by providing a more spacious and adaptable environment, ensuring that all essential equipment and personnel are accommodated comfortably. This space optimization is crucial for the seamless execution of procedures, minimizing potential logistical hurdles, and facilitating the efficient delivery of cardiac care [22].

Optimal patient care: The adaptability of hybrid ORs significantly influences patient care, primarily by expanding procedural possibilities rather than directly dictating anesthetic choices. While the acknowledged benefit of optimizing space in hybrid ORs is valid, attributing the tailoring of anesthetic regimens solely to the physical location within the hybrid OR may be a less substantive assertion. The paramount consideration in delivering optimal patient care is the procedural versatility afforded by the hybrid OR rather than a direct impact on anesthetic choices. The adaptability of this setting enables a broad spectrum of interventions, including minimally invasive surgeries, which can contribute to improved outcomes and reduced postoperative discomfort. However, suggesting that anesthetic customization is exclusively influenced by the hybrid OR's physical location may not be a direct or reasonable correlation. Acknowledging that anesthetic decisions primarily hinge on the specific surgical procedure, patient characteristics, and overall clinical context is crucial. While the hybrid OR provides a dynamic environment for various cardiac interventions, tailoring anesthetic regimens should be grounded in procedural requirements and individual patient needs rather than being uniquely linked to the physical location within the hybrid OR [22].

Single anesthetic for complex conditions: The hybrid OR stands out for its capability to provide comprehensive care to patients with complex cardiac conditions. In cases involving multiple cardiac issues, such as right atrial thrombus, pulmonary artery thrombi, coronary artery disease, and post-infarct ventricular septal rupture, the hybrid OR serves as a versatile solution. It allows for the successful management of these complex conditions in a streamlined manner, showcasing its efficacy in handling intricate cardiac scenarios. The hybrid OR's unique strength lies in its ability to offer integrated care, contributing to the efficiency and cohesiveness of the treatment process for patients with complex cardiac issues. It is important to note that while the term “single anesthetic” may imply a more profound sedation level, the emphasis here is on the holistic management of various cardiac conditions within the hybrid OR setting. This integrated approach enhances efficiency and underscores the comprehensive and patient-centered treatment of complex cardiac conditions. Thus, the hybrid OR's versatility in managing diverse cardiac pathologies is invaluable in providing optimal care to patients with intricate cardiovascular needs [6].

Catheterization Laboratories

An alternative setting for cardiac interventions pre-procedure, intra-procedure, and post-procedure practices: The comprehensive review emphasizes the continuum of practices within the cardiac catheterization laboratory, spanning pre-procedure, intra-procedure, and post-procedure care [23]. This holistic approach thoroughly examines patient management throughout the catheterization process, incorporating critical considerations at each stage to optimize outcomes and patient well-being.

Evolving role: The role of the catheterization laboratory has undergone significant evolution, expanding beyond its traditional boundaries. Incorporating strategies such as venoarterial extracorporeal membrane oxygenation, coronary angiography, and percutaneous coronary intervention, the cath lab has become a dynamic hub for advancing the management of various cardiac conditions [24]. This evolution reflects a commitment to staying at the forefront of technological and procedural advancements in cardiovascular care.

Cardiac arrest management: Managing cardiac arrest within the cath lab presents unique challenges, particularly during resuscitation efforts. The review highlights considerations for alternative options such as mechanical compression devices and invasive percutaneous mechanical circulatory support devices, showcasing a nuanced approach to cardiac arrest management in this specialized environment [25]. Adapting to emerging technologies reflects a commitment to optimizing outcomes in critical situations.

Interventional imaging eco-system: Advances in the interventional imaging eco-system within the cath lab have played a pivotal role in refining cardiac interventions. Co-registration and real-time ultrasound and electromagnetic imaging display enhance precision during procedures, providing clinicians with invaluable insights and contributing to the continuous improvement of interventional techniques [26]. This emphasis on refining imaging technologies underscores a commitment to elevating the quality and efficacy of cardiac interventions.

Operational management: Effective operational management within the cath lab is paramount and involves strategic decision-making concerning resource configuration, adherence to clinical practices, and the assessment of quality and efficiency through registry programs and consultation services [27]. These initiatives demonstrate a holistic approach to optimizing the operational aspects of the catheterization laboratory, ensuring that it functions as a well-coordinated and high-performance center for cardiovascular care. The emphasis on operational excellence aligns with the broader goal of enhancing patient care and advancing interventional techniques within the evolving landscape of the cardiac catheterization laboratory.

Electrophysiology Labs

Managing arrhythmias beyond the OR diagnosing arrhythmias: Electrophysiology labs (EPLs) play a crucial role in diagnosing arrhythmias by employing a range of diagnostic tests to determine the type and severity of these irregular heartbeats. Tests such as electrocardiograms, Holter monitors, event recorders, and echocardiograms provide a comprehensive understanding of the nature of arrhythmias, aiding in their accurate diagnosis [28]. This diagnostic phase is foundational for tailoring effective treatment strategies.

Electrophysiologic studies (EPS): EPS, a diagnostic procedure conducted in EPLs, plays a pivotal role in recording intracardiac signals that may not be evident in surface electrocardiograms. This diagnostic precision is instrumental for identifying the mechanisms underlying cardiac arrhythmias, paving the way for developing targeted and efficacious ablative therapies [29]. The significance of EPS lies in its ability to unveil intricate intracardiac details, contributing substantially to the success of subsequent treatment interventions.

Treatment options: EPLs offer various treatment options for arrhythmias, ranging from pharmaceutical interventions to minimally invasive procedures, electric shock treatments, and implantable devices. The choice of treatment depends on the type and severity of the electrical heartbeat irregularities, highlighting the tailored and comprehensive approach taken in EPLs [28]. These diverse treatment modalities ensure that patients receive personalized care suited to their specific arrhythmic conditions.

Specialized treatment programs: EPLs play a pivotal role in the comprehensive management of arrhythmias, addressing a diverse range of rhythm disorders rather than a narrowly defined category. Within these labs, specialized treatment programs are designed to offer targeted and advanced strategies tailored to specific arrhythmias, including conditions such as atrial fibrillation and ventricular arrhythmias. This approach underscores the nuanced and patient-centered care provided in EPLs, ensuring that individuals with various rhythm disorders receive specialized attention and interventions aligned with the unique characteristics of their specific arrhythmia. The emphasis on comprehensive arrhythmia management reflects the broad scope of conditions addressed within EPLs, showcasing a commitment to delivering tailored and effective care for a diverse array of rhythm disorders [28].

Device clinics: Device clinics within EPLs play a critical role in the long-term maintenance and monitoring of implantable cardiac devices like pacemakers and defibrillators. These clinics ensure that patients with such devices receive ongoing care, monitoring, and necessary adjustments to optimize device performance and patient well-being [28].

Advancements in arrhythmia management: The forward-looking aspect of arrhythmia management involves anticipating gene therapy for arrhythmias, which is expected to enter clinical trials within the next decade. This represents a future frontier in electrophysiology, emphasizing the labs' commitment to staying at the forefront of technological and therapeutic advancements [30]. Electrophysiology labs, through their utilization of the latest technologies and techniques, are pivotal players in the continuous advancement of arrhythmia management. They provide personalized care, contributing to ongoing improvements in patient outcomes and the overall landscape of arrhythmia treatment.

Imaging Modalities in Non-Operating Room Cardiac Anesthesia

Transesophageal echocardiography (TEE): TEE is a crucial imaging modality in non-operating cardiac anesthesia, offering real-time, high-resolution imaging of the heart and great vessels. Its utility is particularly evident in guiding intricate procedures such as atrial septal defect closures, left atrial appendage occlusions, and mitral valve interventions [31]. TEE's ability to provide detailed and dynamic images enhances precision and safety during these procedures, improving patient outcomes.

Transthoracic echocardiography (TTE): TTE serves as another vital imaging modality in non-OR settings, assessing cardiac structure and function. Its noninvasive nature and rapid applicability make it invaluable for providing swift cardiac function evaluations during various procedures [32]. TTE enhances the anesthesiologist's ability to monitor and respond to real-time changes in cardiac dynamics, optimizing patient care during interventions outside the OR.

Fluoroscopy and angiography: Commonly employed in interventional cardiology and cardiac catheterization labs, fluoroscopy and angiography are instrumental in visualizing coronary arteries, cardiac chambers, and major vessels during procedures like percutaneous coronary interventions and structural heart interventions [20]. These imaging techniques provide real-time guidance, aiding in precise catheter navigation and device placement, contributing to the success and safety of interventional procedures.

Electrocardiography (ECG): ECG monitoring is indispensable for continuously assessing cardiac rhythm and conduction during non-OR cardiac procedures. It is a critical tool in detecting arrhythmias and ischemic changes, allowing for prompt intervention and adjustment of anesthesia delivery as needed [31]. ECG monitoring ensures the maintenance of cardiac stability throughout various interventions.

Intraoperative imaging: The role of intraoperative imaging techniques, specifically fluoroscopy, and angiography, is integral in guiding the placement of cardiac devices and assessing their function during non-operating room (NORA) procedures [20]. This dynamic imaging support allows anesthesia providers to adapt their approach based on immediate feedback, contributing to the overall precision and success of various interventional and diagnostic procedures conducted in NORA settings. As discussed previously under a different heading, fluoroscopy, and angiography warrant emphasis due to their pivotal role in real-time visualization. These imaging modalities not only guide the placement of cardiac devices but also assist in assessing their functionality, ensuring the safe and effective delivery of anesthesia in NORA cardiac settings.

Patient selection and evaluation

Criteria for Identifying Suitable Candidates

Patient selection and evaluation constitute pivotal aspects of anesthesia care. The anesthesiologist is tasked with thoroughly examining the patient's medical history, physical condition, and available cardiac and pulmonary function tests during the preoperative consultation [33]. Furthermore, a meticulous preoperative assessment, conducted well before the scheduled surgery, allows for collaboration with the transplant specialist responsible for the patient's care [34]. In the case of geriatric patients undergoing cardiac surgery, an anesthesia geriatric evaluation becomes instrumental in selecting patients who will benefit from preoperative multidisciplinary team care [35].

The field of NORA is experiencing significant growth, and the delivery of anesthesia in NORA settings should adhere to the same exacting standards as those upheld in the operating room [20]. A comprehensive review of the literature in cardiac anesthesiology has identified noteworthy articles poised to influence current and future clinical practices [36]. In essence, the patient selection and evaluation process demand a thorough and inclusive approach, considering the patient's medical history, present health condition, and the specifics of the intended surgical procedure.

Preoperative Assessment in Non-Operating Room Environments

Conducting preoperative assessments in non-OR environments poses distinctive challenges, including the coordination of resources, time constraints, and a potential lack of familiarity with anesthesia protocols. An article focused on NORA underscores the necessity for a comprehensive preoperative evaluation, acknowledging that it may be less extensive than assessments for patients in traditional operating rooms [37,38]. Personnel working in non-OR settings might not be well-versed in anesthesia-related protocols, and the absence of a dedicated preoperative clinic can impede the thorough evaluation of patient-specific comorbidities [19].

Despite these challenges, implementing protocols and fostering interdisciplinary teamwork are emphasized to streamline safe and efficient procedural care in NORA settings [19]. Therefore, while preoperative assessments in non-OR environments may present heightened complexity, ensuring the safety and well-being of patients undergoing procedures outside the conventional OR remains paramount. The emphasis on protocols and collaboration serves as a strategic approach to navigating these challenges and upholding high standards of patient care in diverse procedural settings.

Special Considerations and Risk Stratification

Addressing special considerations and implementing effective risk stratification in NORA settings is imperative, given the distinctive challenges inherent in these environments. The absence of stringent preoperative check-in procedures, time constraints for thorough preoperative assessments, and the imperative for maintaining high-quality standards in NORA settings underscore the need for specialized approaches [20,39]. To address these challenges, an integrated methodology is recommended to facilitate risk stratification, risk reduction, and optimization of care well before the scheduled procedure [20]. A comprehensive review accentuates the importance of considering unique factors during patient selection and throughout the preoperative, intraoperative, and postoperative phases in NORA settings [20]. This holistic approach recognizes the necessity for tailored considerations at each stage to ensure the safety and success of procedures conducted outside the conventional OR.

Furthermore, the role of an anesthesia geriatric evaluation is underscored as an invaluable tool for guiding patient selection, particularly in the context of preoperative multidisciplinary team care in cardiac surgery [35]. This specialized evaluation recognizes the unique needs of geriatric patients, contributing to a more nuanced and personalized approach to their care. The distinctive nature of NORA settings demands a meticulous focus on thorough risk assessment, individualized patient selection, and the implementation of specialized care protocols. These measures are essential to safeguard the safety, well-being, and optimal outcomes of patients undergoing procedures in environments outside the traditional OR.

Anesthetic management in non-operating room settings

Drug Selection and Administration

Patient-specific factors: The selection of anesthetic drugs is intricately tied to patient-specific factors, encompassing considerations such as age, weight, existing comorbidities, and the patient's history of prior medications. Anesthesiologists must meticulously evaluate these individual factors to tailor the administration of anesthetic agents, ensuring not only the efficacy of the anesthesia but, more importantly, the safety of the patient and the achievement of optimal outcomes [6].

Surgical procedure: The nature of the procedure and the patient's overall medical condition play pivotal roles in guiding the anesthesiologist's choice of appropriate anesthetic drugs. For instance, in cardiac surgery, the patient's ischemic risk profile may influence the selection of specific anesthetic agents. This consideration highlights the importance of aligning the choice of anesthesia with the unique demands and intricacies of the surgical intervention [6].

Anesthetic goals: Anesthesiologists aspire to attain a delicate equilibrium between patient comfort, hemodynamic stability, and the induction of amnesia during surgery. Achieving these goals often necessitates a judicious combination of drugs, incorporating opioids for analgesia, muscle relaxants for controlled patient movement, and inhaled anesthetics for the modulation of consciousness levels. This multifaceted approach ensures the patient experiences optimal conditions throughout the surgical procedure [6].

Pharmacokinetics and pharmacodynamics: Anesthesiologists must deeply understand the pharmacokinetic and pharmacodynamic properties of various anesthetic drugs. This knowledge is crucial for accurate dosing and vigilant monitoring during surgery, ensuring that the drugs are administered at appropriate levels to achieve the desired anesthetic effect while minimizing the risk of adverse reactions. Familiarity with these pharmacological aspects is fundamental for maintaining patient safety and the overall success of anesthetic management [6].

Drug interactions: The administration of preoperative medications, such as antibiotics or anticoagulants, introduces the potential for interactions with anesthetic drugs, which can significantly impact patient outcomes. Anesthesiologists must exercise keen awareness of these potential interactions, as they can influence the efficacy and safety of the anesthetic plan. Adjustments to the anesthetic approach may be necessary to mitigate the risks associated with drug interactions, ensuring optimal patient care and outcomes during surgery [6].

Emerging research: Ongoing research in anesthetic drugs and techniques plays a pivotal role in shaping advancements within anesthesia practice. Noteworthy examples encompass a broad spectrum of investigations, including recent efforts related to NorA inhibitors. It's essential to clarify that the mention of NorA inhibitors in this context is unrelated to their conventional use as antibacterial agents. Recent studies have delved into exploring NorA inhibitors, primarily recognized for their antibacterial properties [40]. Additionally, novel anesthetic drugs are being developed to target the *Staphylococcus aureus* multidrug NorA efflux pump [41]. This emerging research holds promise for the future of anesthesia, with potential implications for patient care and outcomes. Staying informed about these developments is crucial for anesthesiologists, allowing them to integrate innovative approaches into their practice, thereby improving the safety and efficacy of anesthesia administration.

Monitoring Techniques and Equipment

Patient monitoring during anesthesia is a fundamental aspect of anesthesia care, dedicated to ensuring the safety and well-being of individuals undergoing procedures. Standard monitors routinely employed during anesthesia encompass a pulse oximeter, electrocardiography, a noninvasive blood pressure device, and a temperature monitor [42]. Moreover, essential components for patient safety include measuring end-tidal carbon dioxide (ETCO_2_), monitoring inspired oxygen concentration, and implementing alarms for low oxygen concentration and ventilator disconnect [42].

The American Society of Anesthesiologists (ASA) has established standards for basic anesthetic monitoring, forming the foundational framework for the minimal monitoring required throughout all anesthesia care. These standards underscore the continuous presence of a qualified anesthesia provider and advocate for diverse monitoring devices to ensure proper oxygenation, ventilation, and patient assessment [43,44]. The recommended minimum monitoring elements for anesthesia encompass pulse oximetry, noninvasive blood pressure monitoring, electrocardiography, and ETCO_2_, among other parameters [45]. Capnography, ventilation monitoring, and the uninterrupted presence of qualified anesthesia personnel are also emphasized in these standards [44,45].

Team Collaboration and Communication

Successful anesthesia care hinges on effective team collaboration and communication, as anesthesiologists operate within interdisciplinary teams comprising surgeons, nurses, and various healthcare professionals. The paramount goal is to ensure patient safety and achieve optimal outcomes. Collaborative communication within this context involves fostering open, honest, and respectful dialogue among team members. This entails creating an environment where individuals feel empowered to voice their opinions and contribute ideas [46,47].

Anesthesiologists play a crucial role in facilitating effective communication within the team. This involves using appropriate communication tools, understanding team members' working and communication styles, promoting open feedback, practicing active listening, and securing team buy-in for any proposed changes [46]. Additionally, by cultivating a culture of collaborative communication and exemplifying it as leaders, anesthesiologists can significantly enhance project outcomes and expedite goal attainment [46].

Open and truthful communication is the linchpin of effective team collaboration. The more team members feel encouraged to express themselves, the more robust the collaboration becomes [48]. Anesthesiologists must also champion transparency, sharing knowledge, insights, and resources while leading by example to foster creativity and a communal working environment [48]. In essence, effective team collaboration and communication stand as indispensable elements for successful anesthesia care. Anesthesiologists are responsible for nurturing a culture of collaborative communication to ensure patient safety and achieve optimal outcomes in the dynamic and interdisciplinary field of anesthesia.

Complications and contingency planning

Recognizing and Managing Complications in Out-of-OR Procedures

The administration of anesthesia outside the traditional OR (NORA) introduces potential patient safety risks, attributable to problematic case schedules, extended internal commutes between the main OR and procedure suites, and limited access to tools typically available in the operating room [49]. To mitigate errors and prioritize patient safety, it is crucial to establish structured communication channels among the patient, surgeons, and other healthcare team members. This begins with implementing a formal procedure for the final confirmation of the correct patient and surgical site, often called a “time-out” [50,51]. Acknowledging the inevitability of complications, a well-prepared anesthesia team should be able to anticipate and manage them proactively and effectively [51]. Successful conflict resolution during complications necessitates cultivating mutual respect between surgeons and anesthesiologists. This involves attentive listening, careful consideration of the issues at hand, recognizing differences in perspectives, and acknowledging the emotional aspects inherent in disagreements [51].

Emergency Protocols and Rapid Response Teams

Rapid response teams (RRTs) or medical emergency teams (METs) are specialized groups of healthcare clinicians strategically assembled to provide prompt critical-care expertise in response to serious clinical situations [52]. The primary objective of RRTs is to avert intensive care unit transfers, cardiac arrests, or fatalities by swiftly evaluating and treating patients exhibiting signs of imminent clinical deterioration [53]. Typically, RRTs are nurse-led, whereas METs are led by a physician, often an intensivist, with both teams equipped with critical care skills necessary for rapid assessment and response [53].

The establishment of RRTs or METs within hospital settings originated to address the challenge of “failure to rescue,” a situation often rooted in planning deficiencies encompassing assessments, treatments, and goals [53]. RRTs are trained to employ effective communication methods, often utilizing SBAR (Situation, Background, Assessment, Recommendation), and follow a structured documentation form [53]. The widespread adoption of RRTs is attributed to their proven effectiveness in reducing hospital cardiopulmonary arrests, establishing them as a crucial patient safety intervention [54].

Postoperative Care and Follow-Up

Postoperative care is the specialized care administered following a surgical procedure, commencing immediately after surgery and potentially extending beyond the patient's discharge. This tailored care is contingent upon the nature of the surgery and the patient's health history, typically encompassing facets such as pain management, wound care, and comprehensive education regarding potential complications [55]. The significance of postoperative follow-up cannot be overstated, particularly for surgical patients with substantial comorbidities, as it facilitates vigilant monitoring and early identification of any complications that may arise [56].

In specific cases, such as colorectal cancer surgery, frequent follow-up visits within the initial two years are imperative to monitor progress and detect any potential recurrence of cancer. Subsequent follow-ups, although less frequent, are advisable after five years to identify new polyps [57]. Providing optimal postoperative care, including encouraging early ambulation, meticulous wound care, and vigilant monitoring for complications, is pivotal in mitigating the risk of postoperative complications [58]. Furthermore, in outpatient surgery, adherence to stringent guidelines and implementing a robust control system are indispensable to ensure patient safety and promptly detect any postoperative issues [59]. In summary, postoperative care and follow-up procedures play an integral role in facilitating recovery, promptly identifying complications, and ultimately contributing to achieving the best possible outcomes for surgical patients.

Training and education for cardiac anesthesia in non-operating room settings

Specialized Training Programs and Courses

Numerous specialized training programs and courses are available for individuals pursuing cardiac anesthesia careers. The American Board of Anesthesiology (ABA) facilitates special training programs that enable residents to incorporate up to an additional year of research or specialized training beyond the standard 12 [60]. The University of Cincinnati College of Medicine provides a dedicated curriculum in cardiothoracic anesthesiology, offering residents a comprehensive continuum of education and hands-on experience in this subspecialty [61].

The ABA further extends its offerings with specialized training programs focusing on critical care medicine, pain medicine, adult cardiothoracic anesthesiology, hospice and palliative medicine, sleep medicine, or pediatric anesthesia [62]. Stanford University School of Medicine presents the Adult Cardiothoracic Anesthesiology (ACTA) Fellowship, a rigorous one-year clinical training program designed to equip graduates for leadership roles in both clinical and academic settings [63]. For residents venturing into the practice of anesthesia outside the traditional OR, it is imperative to establish a strong foundation in anesthetic training [64]. The diverse range of specialized programs available reflects the commitment to providing tailored education and hands-on experience in cardiac anesthesia, ensuring that practitioners are well-prepared for the complexities of their chosen subspecialty.

Simulation and Virtual Reality in Skill Development

Simulation and virtual reality (VR) are gaining prominence in skill development, particularly within cardiac anesthesia. VR technology has been harnessed for various training applications, including simulation-based training facilitated by VR technologies [65]. Research indicates that, on average, VR training surpasses traditional training methods in fostering the technical skills of students [66]. VR simulation presents an innovative approach to training, addressing technical and non-technical skill acquisition [67].

VR in skill development allows learners to practice skills, comprehend intricate concepts, and navigate diverse work scenarios within an interactive and realistic training environment [68]. In the specialized field of cardiac anesthesia, VR simulation emerges as a valuable tool, offering a safe and controlled setting for trainees to hone their skills and engage in hands-on practice of procedures [68]. The incorporation of VR in skill development not only enhances the effectiveness of training but also provides a dynamic and immersive learning experience for individuals in cardiac anesthesia.

Continuous Professional Development and Certification

Continuous professional development and certification in cardiac anesthesia necessitate ongoing education and training to both uphold and advance skills. For instance, the ABA mandates that anesthesiologists accrue 125 Continuing Medical Education (CME) credits every five years to sustain their certification [69]. Recognizing the specialized expertise in Adult Cardiac Anesthesiology (ACA), the ABA has introduced a new board certification to acknowledge professionals excelling in this area [70].

Embracing innovative approaches to training, VR simulation has demonstrated efficacy in developing technical skills [66,67]. This technology offers a progressive avenue for training and skill acquisition, providing professionals with a secure and controlled environment to refine their expertise. In cardiac anesthesia, where precision and proficiency are paramount, VR simulation proves particularly relevant [67]. As such, continuous professional development in cardiac anesthesia encompasses pursuing CME credits, specialized certifications, and integrating cutting-edge training modalities like VR simulation, ensuring professionals stay current and further enhance their technical skills.

## Conclusions

In conclusion, this review has underscored the transformative shift in cardiac anesthesia from its traditional OR roots to contemporary approaches outside these confines. The findings emphasize the pivotal role of non-OR environments, such as hybrid operating rooms and catheterization laboratories, in reshaping the landscape of cardiovascular interventions. The review has elucidated key considerations, including patient selection, specialized anesthetic management, and the integration of advanced imaging modalities, all of which contribute to the success of interventions in these diverse settings. The implications for the future of cardiac anesthesia are profound, with the potential to optimize patient outcomes, reduce complications, and enhance resource efficiency. As we look ahead, the field is poised for continued evolution, marked by the integration of cutting-edge technologies and a commitment to personalized, patient-centered care. Encouraging further research and fostering collaborative efforts among healthcare professionals, researchers, and industry stakeholders will be paramount in navigating the complexities of contemporary cardiac interventions, ensuring ongoing advancements, and ultimately improving the quality of care for individuals undergoing cardiovascular procedures.
